# Public health and English local government: historical perspectives on the impact of ‘returning home’

**DOI:** 10.1093/pubmed/fdt131

**Published:** 2014-01-27

**Authors:** Martin Gorsky, Karen Lock, Sue Hogarth

**Affiliations:** Faculty of Public Health and Policy, London School of Hygiene and Tropical Medicine, 15–17 Tavistock Place, London WC1H 9SH,UK

**Keywords:** public health, government and law, health services

## Abstract

This article uses history to stimulate reflection on the present opportunities and challenges for public health practice in English local government. Its motivation is the paradox that despite Department of Health policy-makers’ allusions to ‘a long and proud history’ and ‘returning public health home’ there has been no serious discussion of that past local government experience and what we might learn from it. The article begins with a short resumé of the achievements of Victorian public health in its municipal location, and then considers the extensive responsibilities that it developed for environmental, preventive and health services by the mid-twentieth century. The main section discusses the early NHS, explaining why historians see the era as one of decline for the speciality of public health, leading to the reform of 1974, which saw the removal from local government and the abolition of the Medical Officer of Health role. Our discussion focuses on challenges faced before 1974 which raise organizational and political issues relevant to local councils today as they embed new public health teams. These include the themes of leadership, funding, integrated service delivery, communication and above all the need for a coherent vision and rationale for public health action in local authorities.

The long history of public health in local government offers a resource for today's policy-makers as they seek to maximize opportunities for reducing health inequalities during the current public health transition.

The Health and Social Care Act 2012 has reconfigured the structure of English public health, moving significant powers to local government. This is presented by the Department of Health (DH) as reviving ‘a long and proud history’ by ‘returning public health home’ to its pre-1974 location.^1^ Building on cross-party support for furthering localism, policymakers argue that local government is best placed to ‘shape environments’ and to reduce health inequalities.^2,3^

These themes are not new to National Health Service (NHS) politics. Indeed, some were present from the service's foundation, when, on grounds of efficiency and equity, Aneurin Bevan overruled Labour Cabinet members who favoured an entirely local government-based NHS.^4^ The organizational model established in 1948 was a compromise: limited public health functions were retained by local authorities, each under a Medical Officer of Health (MOH), while their pre-existing hospital and clinic services were transferred to new NHS bodies. Public health's location then remained stable until 1974 when NHS reorganization removed all but environmental health duties to health authorities, and terminated the MOH role. Although a public health presence was maintained by new ‘community physicians’ in the restructured NHS, many characterize the period as one of ‘decline’.^5^ Only after the 1988 Acheson Report,^6^ following concern generated by HIV/AIDS and food poisoning scandals, did a revitalization of the public health function in England occur.

Public health's previous move out of local government has not been discussed during current policy development, excepting an oblique DH observation that the context has ‘changed hugely since 1974’.^1^ In this paper, we begin by exploring the history of public health in local government between its Victorian roots and the start of the NHS. We then examine its performance between 1948 and 1974, when there were various structural parallels with the present. We conclude with reflections on how this history might inform us during the present transition for English public health.

## Public health before the NHS

Health and social welfare functions in English local government extend back at least to Tudor times, when parishes were given responsibilities for poverty and the environment. However, the modern public health function is best traced to the early nineteenth century, when government responded to social impacts of economic development. Gradual improvements in life expectancy, which began as growth accelerated, stalled by the 1820s. This reflected the impact of rapid industrialization and urbanization which led to poor neighbourhood and housing environments, occupational health risks in unregulated workplaces and heightened prevalence of communicable diseases, including tuberculosis, typhoid and cholera.

**Fig. 1:**
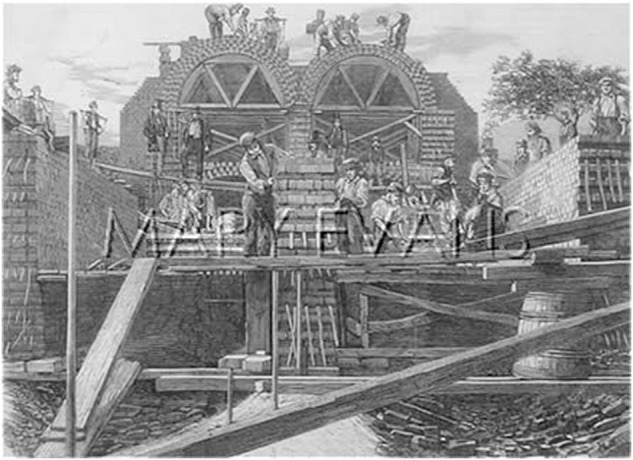
London Sewer Construction in Bow, 1859. Copyright Illustrated London News/Mary Evans Picture Library, ref 10010475. The public health service in its Victorian phase is associated with major capital projects, authorized and resourced by elected councils. In Thomas McKeown's famous account of the modern rise of population, he calculated that 33% of the mortality decline between 1848/54 and 1901 was attributable to water-borne diseases amenable to such interventions.^8^

Social reform came in steps.^7^ Legislation introduced a New Poor Law (creating workhouses designed to deter dependency) and established elected municipal governments with powers of taxation. In 1848, councils were empowered to appoint an MOH and improve urban hygiene; Public Health Acts in the 1870s further strengthened local departments. In 1842, Edwin Chadwick's report convinced many of the need for sanitary investment, even though the germ theory was not yet understood (Fig. 1). From 1837, civil registration of deaths gave local policymakers data on rates of mortality by age, sex, cause and place. Pasteurian bacteriology legitimized other extensions of local public health duties, which by 1900 included drainage, sanitation, safe water supplies, street cleansing, disinfection, disease notification and isolation, food safety, smallpox vaccination and institutional care principally for ‘lunatics’ and infirm older people. Between 1900 and 1929, eugenics and militarism fuelled population health concerns that conferred new local responsibilities for maternal and child welfare, health visiting, school medicine, venereal diseases and learning disabilities, while the ‘homes fit for heroes’ agenda involved health departments in slum clearance.^8,9^ In the 1930s, stigmatizing aspects of the Poor Law were diminished, with ex-workhouses used as municipal general hospitals or institutions for older people. By then, the social and health responsibilities of local government had expanded to include a wider range of actions than is covered by current NHS public health practice.

**Fig. 2:**
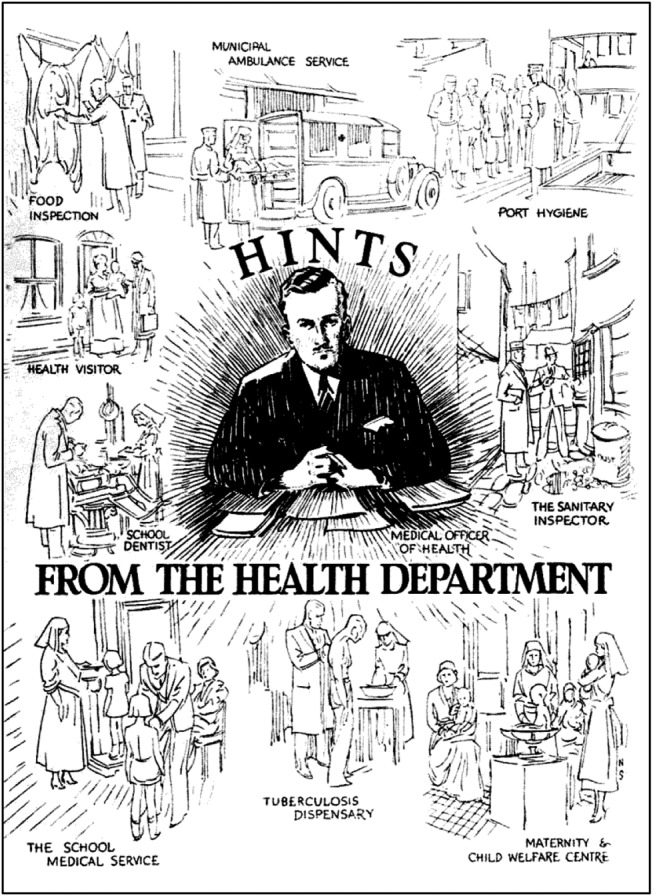
‘Hints from the Health Department’, Leaflet, nd. Wellcome Library, London, reference CMAC SA/SMO/R.4/13. The local executive power of the MOH, the town's figurehead for public health, is projected here as paternalist reassurance. Around him are represented health duties integrated with other municipal activities including housing, education, social care, regulation of commerce and business, as well as cure and prevention.

Historians today are wary of characterizing this as a ‘golden age’ for public health.^10^ Despite increasing life expectancy, from 40 years in the 1850s to 60.8 in 1930,^11^ serious health inequalities and high maternal mortality rates persisted. Many factors, such as improved nutrition, female education and smaller families, also contributed to better population health. Yet evidence suggests at least some of this improvement should be attributed to the local public health service, with its broad range of environmental and medical services integrated under the MOH (Fig. 2).

## Public health in early NHS (1948–74)

With the establishment of the NHS, the size and scope of public health departments were substantially reduced, as Regional Hospital Boards took control of hospitals, and universal access to primary care was introduced. Bevan's concern had been that the capacity of local government was too uneven to ‘universalise the best’ in the way he envisaged for care within the NHS. Nonetheless, there was official priority given to ‘… the promotion of good health rather than only the treatment of bad’.^12^

Between 1948 and 1974, local government health departments typically retained a separate identity, with a staff of environmental and clinical medical officers, and health visitors and home nurses to supply community services. The framework for local delivery outlined at the NHS's creation bore many similarities to that enacted for public health in 2012.^13,14^ There was a public health leadership role, today the Director of Public Health (DPH), then the MOH; there was an obligation to produce an annual report dealing with population health and services, just as today; there was a range of specified duties, spanning health education, prevention and treatment of diseases, maternity and child welfare services, immunization programmes and community care for older people; today, these still include health promotion, prevention, emergency responses and environmental health improvement.^15^ Historically, local government's environmental roles (housing, slum clearance, inspection of factories, air quality etc.) conferred a major role in ‘shaping local places’. Today, local public and environmental health departments continue to be involved not only in food and air quality, but also housing, urban planning, transport and other policies that affect environmental and social determinants of health. In both the early and current NHS, voluntary organizations were incorporated as providers of community services. Integration of local government with NHS functions has always been vital, and like earlier MOsH, the DsPH must ensure joint working, whether as representatives on NHS bodies, or local authority leads on joint processes, such as joint strategic need assessments. Only public engagement, underpinned today by Local Healthwatch Organizations, differed, in that existing democratic processes were previously thought sufficient.

Why then were these restructured local government public health departments so short-lived? Their abolition came in the 1974 NHS reform, with the creation of a hierarchy of regional and local health authorities and the disappearance of the MOH role. Declining morbidity and mortality from infectious diseases meant that health concerns lost their importance within environmental and housing policy. Local government's public health role was also eroded by an expanding welfare state.^16,17^ GPs increasingly took over maternal and child healthcare, with new primary care practices that incorporated enhanced community services including health visiting and home nursing. Then, following the Seebohm Report of 1968, social care was removed from public health's remit and conferred on Social Services Departments, staffed by a new profession: social workers.

Another structural challenge faced by local public health was integration with the NHS. A major problem area surrounded the care of older people with complex needs spanning the health/social care boundary. In an atmosphere of resource constraint, challenges to joint working emerged, with NHS leaders complaining about ‘bed-blocking’ and local authorities fearful of cost shunting as they struggled to provide residential care.^18^ This was one driver behind the decision to introduce new health authorities in 1974 that would coordinate hospital, primary and community care.

Finally, historians suggest that local public health departments failed to establish a coherent philosophical and disciplinary underpinning for their work in an era of epidemiological transition. Most MOsH continued to focus on tackling communicable diseases and health service administration.^17^ This meant a divergence between ‘service’ public health practiced by MOsH and academic ‘social medicine’, which sought to reorient the field towards chronic disease epidemiology and to social determinants of health.^19^ In the inter-war years, MOsH were criticized for neglecting their ‘watchdog’ role over the health of the poor, and being unwilling to court political controversy. Thus, the health advocacy and behaviour change agenda was increasingly set elsewhere. For example, local health education on tobacco consumption was limited, and the issue increasingly taken up by national government and voluntary organizations.^14,20^ Nor was a broader environmental health agenda developed until WHO's Healthy Cities initiative legitimized concerns like transport, air quality and climate as part of health policy.

## Implications for current local public health practice

So, what issues does this history raise for public health in local councils today?

### Leadership

**Fig. 3:**
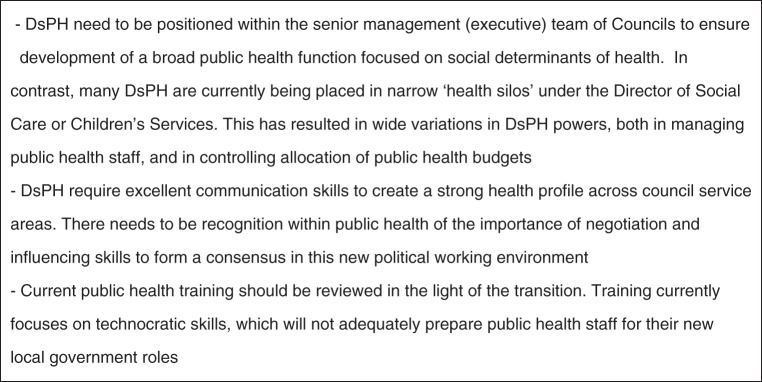
Issues of public health leadership in transition to local government.

A secure executive position independent of vested interests was the historic basis for MOsH in sustaining political and community relationships and the adoption, if necessary, of politically controversial positions. In the current transition, many factors will influence the impact of public health in councils (Fig. 3), but most important is likely to be the seniority and position of the DPH role within the council. Despite DH guidance that DsPH should be part of the senior management structure, examples already exist of DsPH appointed subordinate to other directors with public health teams part of other directorates including adult and children services.^21,22^

### Funding

Bevan's concern was that devolved power did not lead to virtuous localism but rather to spatial unevenness, with inadequate performance in poorly resourced areas. The current focus on localism makes this still a continued threat. Public health budgets have been initially ring-fenced, with a recent increase in funding for those local authorities with the worst socioeconomic conditions.^23^ Removal of ring-fencing is being demanded to allow councils flexibility to mitigate the impact of further local government budget cuts of 10%; announced in the spending review for 2015–16, on the top of the 28%; funding cuts for 2011–15.^24^ Under continuing austerity, it will be vital to ensure that the public health funds are not dissipated by other local government priorities.

### Public health service delivery

For the first time since 1974, councils will be required to commission new public health services traditionally delivered by the NHS, including sexual health and individual health checks. Historically, local councils are well placed to commission and deliver these community services. DPHs will also need to ensure public health priorities remain embedded across other local organizations, such as primary care under the clinical commissioning groups (CCGs), and improving the strategic coordination of commissioning across NHS, social care and related services through Health and Wellbeing Boards. The main mechanisms to foster local joint working include the development of joint strategic need assessments and health and wellbeing strategies. DPHs also have a role in monitoring national bodies delivering PH services locally, for example, the role of Public Health England delivering screening and immunizations. The importance of these different mechanisms, and the scale of the coordination task for public health, needs to be acknowledged and prioritized by local council and health leaders if the failings of integrated working in the early NHS are not to be repeated. In addition, public health practitioners face the challenge of developing or strengthening relationships internally, across a range of local government directorates, from housing, planning and transport, to alcohol licensing, to better tackle the health inequalities agenda within social and environmental policy.

### Engaging councillors and the public

Historically, the annual report was the opportunity for MOsH to make public their review of local population health needs, and set the agenda for health policy, services and resources. Since their post-Acheson reinvention, researchers have discovered both good and bad reporting practice.^25^ It will be important to consider how to maximize the impact of the statutory DsPH annual reports within a democratically elected organization, while learning to maximize the use of local consultation strategies and new communication approaches to engage local public and political support.

### A local public health vision

**Fig. 4:**
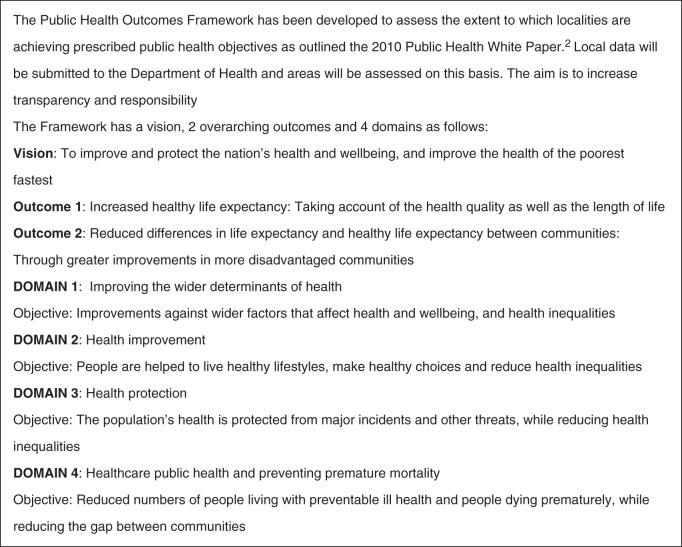
Public Health Outcomes Framework.^30,2^ Local data will be submitted to the DH and areas will be assessed on this basis. The aim is to increase transparency and responsibility. The Framework has a vision, two overarching outcomes and four domains as follows: Vision: to improve and protect the nation's health and wellbeing, and improve the health of the poorest fastest. Outcome 1: increased healthy life expectancy: taking account of the health quality as well as the length of life. Outcome 2: reduced differences in life expectancy and healthy life expectancy between communities: through greater improvements in more disadvantaged communities. DOMAIN 1: improving the wider determinants of health. Objective: improvements against wider factors that affect health and wellbeing, and health inequalities. DOMAIN 2: health improvement. Objective: people are helped to live healthy lifestyles, make healthy choices and reduce health inequalities. DOMAIN 3: health protection. Objective: the population's health is protected from major incidents and other threats, while reducing health inequalities. DOMAIN 4: healthcare public health and preventing premature mortality. Objective: reduced numbers of people living with preventable ill health and people dying prematurely, while reducing the gap between communities.?>

National politicians fully accept that prevention is a health priority (Fig. 4), if the cost of the NHS is to remain sustainable and if health inequalities are to be reduced.^26,27^ Indeed, in contrast to their more cautious MOH predecessors, today's DsPH are mandated in the new Act to tackle inequalities of access and outcomes.^15^ The overriding lesson from the pre-1974 era is that a coherent rationale matters for public health. This needs to draw together both public health practice and research, and provide a basis for political action to address today's priorities. Yet achieving this will not be easy, given the enduring political rhetoric which focuses on changing ‘individual behaviour’ and defends personal choices over regulation and ‘nannying’.^2,28^ Many public health professionals counter that a successful strategy also ‘… requires the Government to do what only it can do; enabling and supporting the efforts of society to … address those barriers … that prevent people from making the healthier choice’.^29^ The challenge for public health in local government today, as historically, is to create a compelling public health vision for both the public and policymakers which bridges the upstream and downstream policy approaches, and is responsive to the needs of a participatory local democratic structure. This will require more relevant indicators to monitor local public health progress and success, which will need to respond to local political priorities as well as the DH's Public Health Outcomes Framework (Fig. 4).

## Authors’ contributions

M.G. conducted the historical research on the role of public health in local government. K.L. and S.H. contributed analysis of the current transition of public health functions from primary care trusts to local government. M.G., K.L. and S.H. all contributed to writing. M.G. is a historian with expertise in NHS and local government public health services. K.L. is a PH academic and Honorary Consultant in Public Health at Public Health England, with experience of public health practice in a range of English organizations. S.H. is a senior public health registrar who has worked in the NHS, including 3 Inner London primary care trusts, since 2005.

## Funding

Martin Gorsky was funded by a Wellcome Trust 5-year award, ref. 088670, ‘Public health and health services in postwar Britain’; Karen Lock was supported by the National Institute for Health Research (NIHR)'s School for Public Health Research (SPHR). The views expressed are those of the author(s) and not necessarily those of the NHS, the NIHR or the Department of Health. Funding to pay the Open Access publication charges for this article was provided by the Wellcome Trust.
